# The effects of smoking on obesity: evidence from Indonesian panel data

**DOI:** 10.1186/s12971-015-0064-5

**Published:** 2015-11-26

**Authors:** Kitae Sohn

**Affiliations:** Department of Economics, Konkuk University, 120 Neungdong-ro, Gwangjin-gu, Seoul 05029 South Korea

**Keywords:** Smoking, Weight, Obesity, Indonesia, Causality, Panel data, Fixed effects model

## Abstract

**Background:**

It has been known that smoking is negatively related to weight-related outcomes. However, it has been difficult to determine whether the relationship is causal, and if so, how strong it is. We attempted to estimate the approximately causal effects of smoking on weight, body mass index (BMI), and obesity.

**Methods:**

The Indonesian Family Life Survey provided a sample of over 9000 men aged 15–55 years—each of them was observed in 1993, 1997, 2000 and 2007. The preferred method was a fixed effects model; that is, we related changes in smoking status or smoking intensity to changes in weight-related outcomes, while controlling for time-varying covariates. We also compared these results to those estimated by ordinary least squares and assessed the importance of controlling for time invariant individual heterogeneity.

**Results:**

Although the effects of smoking were precisely estimated in a statistical sense, their size was minuscule: a quitter would gain weight by at most 1 kg, or a smoker would lose weight by the same amount. The results were similar for BMI and obesity. When we did not control for time invariant individual heterogeneity, the size of the relationship was overestimated at least three times.

**Conclusions:**

Smoking exerted little influence on weight, and it was important to control for bias stemming from time invariant individual heterogeneity.

## Background

Smoking and obesity are two of the greatest health hazards in the world, so a large body of research has investigated the causes and consequences of smoking and obesity. Recently, attention has been paid to the connection between smoking and obesity [1]. It has been argued that smoking increases physical metabolism and reduces the consumption of sweet food; hence, smoking is believed to be related to lower body weight and reduced obesity [2, 3]. Although there is much evidence to support the negative relationship between smoking and weight-related outcomes, almost all studies are based on findings of correlation rather than causation. In contrast to these studies, some health economists have debated the *causal* effects of smoking on weight-related outcomes [4–9]. They typically exploited instrumental variables (IVs), that is, variables that are correlated with smoking but uncorrelated with weight-related outcomes (referred to as exclusion restrictions). Examples include state cigarette costs in the US. Medical researchers generally do not use IVs, but IVs provide an effective means to tease out causality when double-blind, randomized, placebo-controlled trials are unavailable, as in this case. Therefore, health economists are keen to use IVs, whenever plausible IVs are available. Nevertheless, the small number of studies in health economics demonstrated that it was difficult to find plausible IVs, and the results were sensitive to the inclusion and construction of some covariates and the stratification of the samples.

Despite the growing interest in the issue, relatively little attention has been paid to it in the developing world. Considering the sheer number of and the rate of increase in the number of smokers there, however, the developing world deserves more attention. For example, in 1980, there were 441 million smokers (both sexes combined) in developing countries and 280 million smokers in developed countries. In 2012, the corresponding figures changed to 726 and 242 million [10]. In addition, the number of deaths attributable to tobacco use was estimated to increase from 3.4 to 6.8 million deaths in low- and middle-income countries between 2002 and 2030 but to decrease in high-income countries during the same period [11]. Meanwhile, in 1997, the World Health Organization (WHO) declared that obesity was a global epidemic [12]. With obesity at a global scale, the developing world, thought to be unaffected by obesity, also witnessed an emerging epidemic. The proportion of overweight or obese (body mass index, BMI ≥25) adult women aged 18–49 grew by about 0.7 percentage points per year in 42 developing countries [13].

Furthermore, a developing country offers an interesting opportunity for smoking-related issues because smoking is little discouraged there [14]. Public awareness of the adverse effects of smoking is limited; anti-smoking policies are either absent or poorly enforced, and the advertisement, promotion, and sale of tobacco take place openly and widely, even to minors. Empirical results under these circumstances could provide insight into a situation where restrictions on smoking in the developed world are relaxed. If the relationship between smoking and weight is entirely based on a biological mechanism, these environmental differences between the developed and developing worlds would be inconsequential, and separate studies for developing countries would be redundant. It is, however, possible that some environmental factors, such as the health-consciousness of a population, influence smoking and weight-related outcomes. It is thus of great interest to separately consider developing countries. We aimed to estimate the approximately causal effects of smoking on weight, BMI, and obesity in Indonesia, while controlling for bias stemming from time invariant individual heterogeneity.

## Methods

In 1993, the IFLS started to follow over 22,000 people in 7224 households in 13 provinces (IFLS1), which is representative of 83 % of the 1993 Indonesian population. The IFLS sampling scheme stratified by provinces and then randomly selected 321 enumeration areas (EAs) within each of the 13 provinces and then households within a selected EA. For each household selected, a representative member provided household-level demographic and economic information, and several household members were randomly selected and asked to provide detailed individual information.

Four follow-ups ensued in 1997 (IFLS2), 1998 (IFLS2+), 2000 (IFLS3), and 2007 (IFLS4). IFLS2+ is not publicly available and covers only a quarter of the original respondents for some ad hoc purposes. We thus excluded IFLS2+ and used all the other surveys. Most data were collected by interview, but some anthropometrics (e.g., height and weight) were measured in every survey year by two specially trained nurses. The user’s manual does not mention informed consent or institutional review board approval.

One questionnaire module concerned smoking, from which we extracted information on current smoking status and smoking intensity. Smoking status was determined by the answer to the following question: “Do you still have the habit [smoking] or have you totally quit?” If the answer was “still have,” we considered the respondent a current smoker, and if the answer was “quit” or he had not smoked before, a nonsmoker. We measured smoking intensity by the number of cigarettes smoked per day. For a small number of men who chewed tobacco or smoked a pipe, 1 g was assumed to be equal to one cigarette; the ratio of 0.8 g to one cigarette did not affect the following results (not shown). When we analyzed smoking intensity, we treated nonsmokers in two ways. First, we assigned zero to the number of cigarettes per day and created a dummy variable indicating a non-smoker. Second, we excluded the dummy of non-smoking and used only the continuous variable (i.e., number of cigarettes per day). In these two cases, we grouped smoking intensity as follows to allow nonlinearity in the effects of smoking on weight-related outcomes: 0, 1–9, 10–19 and 20+ cigarettes consumed per day. Smoking is an exclusively male habit in Indonesia: in the raw data of IFLS4, only 2.0 % of women aged 15+ had ever smoked, and 1.6 % were currently smoking. Consequently, we considered only men aged 15–55 years in IFLS1, each of whom was observed in all the survey years.

The longitudinal scheme allowed us to use fixed effects models. Specifically, we employed the following specification to estimate the effects of smoking on weight-related outcomes:

1 $$ {w}_{it}={\beta}_1{S}_{it}+{X}_{it}{\beta}_2+{u}_i+{\varepsilon}_{it}, $$

where *w*_*it*_ refers to individual *i*’s weight-related outcome in year *t*, *S* to current smoking status or smoking intensity, *X* to a vector of time-varying covariates, *β*_1_ and *β*_2_ to coefficients, *u* to any individual characteristics that do not vary over time (i.e., time invariant individual heterogeneity), and *ɛ* to the random error term. Potential correlation of *u* and *S* threatens against arguing *β*_1_ to be causal effects. For example, impatience in *u* may lead to both smoking and low weight. Then, *β*_1_ would be biased upward (in absolute values) because *u* and *S* are positively correlated. On the other hand, health-consciousness in *u* could lead to nonsmoking and low weight. In this case, *β*_1_ would be biased downward. Moreover, heredity contained in *u* is believed to affect both *w* and *S* [15–17]. It is thus empirically compelling to control for *u*, which is a critical confounder; almost all studies in the literature neglected this point. For comparison purposes, we presented results derived from ordinary least squares (OLS) with standard errors clustered at the individual level. Comparisons of the results estimated by fixed effects models and OLS demonstrated the degree of bias in *β*_1_.

One concern for fixed effects models is reverse causality, but reverse causality is likely to be small. This is because although absence of evidence is not the same as evidence of absence, information on smoking as a weight control tool is lacking in Indonesia. Even if such information were as common in Indonesia as in the US, this would not make reverse causality serious because even in the US, where such information abounds, reverse causality occurred only for women [18, 19]. Furthermore, because women are more sensitive to weight in general, some Indonesian women might smoke if they strongly believe that smoking reduces weight. The fact that they rarely smoke suggests that reverse causality is likely to be negligible. Another concern for fixed effects models is bias stemming from omitted time-varying covariates. For example, people care more about health over time, so they smoke less and lose weight during the same period. In this case, time-varying health consciousness drives changes in both smoking status and weight, thereby biasing *β*_1_. As shown in the next section, however, comparisons of results estimated by fixed effects models and OLS suggest that bias in *β*_1_ was upward, and even potentially overestimated *β*_1_ in the fixed effects models was small. Therefore, our main argument that *β*_1_ was small remains valid.

We considered weight, BMI, and obesity for *w*. BMI is not an ideal measure for assessing obesity because it cannot distinguish fat from muscle, and among fat, total fat from abdominal fat, while obesity is concerning mainly because of abdominal fat. Nevertheless, BMI is still widely used, particularly in the social sciences because it is readily available and cost-effective in a large survey. In addition, particularly for Indonesia, BMI is a better predictor of obesity-related diseases than are other anthropometrics such as waist circumference, waist-to-height ratio, and waist-to-hip ratio [20]. Hence, we relied on BMI for the assessment of obesity and used the cutoff for obesity not 30 but 25, following the suggestion by the WHO for Asians [21]. This cutoff is reasonable because only 1.5 % of the sample exceeded a BMI of 30.

*X* included age and its squared term, height, marital status, urban (vs. rural) residence, and earnings. Age, marital status, and earnings were self-reported, height was measured, and urban residence was determined based on administrative information. We controlled for age because it is a basic demographic factor related to both smoking and weight-related outcomes; its squared term was intended to capture a potentially nonlinear relationship between the two. We controlled for height because tall people are generally heavier and height reflects early life conditions [22–29]. We entered marital status because it is another basic demographic factor, but we found that it contributed little to explaining *w*. Earnings refer to salaries or wages for paid employees or net profits for the self-employed, earned during the last month [30–32]. We created a dummy indicating men with no earnings and assigned zero to their natural log of earnings. The idea was that earnings affect weight through smoking and including earnings-related variables would tease out the pure effects of smoking on weight.

In all specifications, we performed Hausman’s specification tests to check whether *u* was correlated with *S* or *X*, that is, whether fixed effects models were more appropriate than random effects models. In all cases, the test rejected the null hypothesis that both models would yield the same coefficients, suggesting that fixed effects models were more appropriate (not shown). The sample size varied depending on the covariates controlled for, but it exceeded 9000. We applied longitudinal weights to make the sample representative. Because the data are publically available, no ethical approval was required.

## Results

### Descriptive statistics

**Fig. 1:**
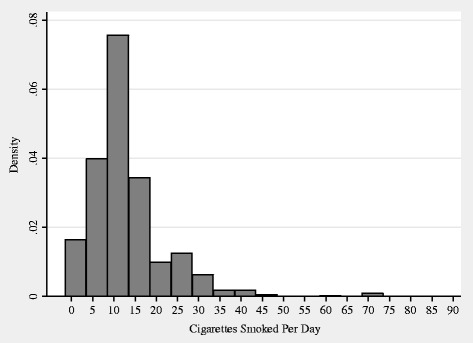
Distribution of Cigarettes Smoked Per Day for Smokers. Notes: We excluded observations with a value of zero. We set the width of bins to five

Table 1 presents descriptive statistics. The mean weight was 55.9 kg, and the mean height was 160.7 cm. Because of the short stature and light weight, the mean BMI was 21.6; consequently, only 13.6 % of the sample reached or exceeded a BMI of 25. About three quarters of men smoked at the time of the interview. Figure 1 shows that most smokers consumed less than a pack per day, which implies that the main action between smoking and weight-related outcomes took place with a smoking intensity of less than a pack per day.

**Table 1:** Descriptive statistics

Continuous variable	Mean (SD)	Discrete variable	Percent
Weight (kg)	55.9 (9.3)	Smoking	71.4
Number of Cigarettes per Day	9.7 (9.3)	Non-Smoking	28.6
Height (cm)	160.7 (5.9)	BMI ≥25	13.6
BMI (kg/m^2^)	21.6 (3.1)	BMI <25	86.4
Age	44.1 (10.2)	With Earnings	82.6
Ln (Monthly Earnings in Rp./1000)	4.39 (2.35)	Without Earnings	17.4
		Never Married	1.5
		Married	96.5
		Separated/Divorced/Widowed	2.0
		Rural Areas	59.4
		Urban Areas	40.6
		0 Cigarette	24.5
		1–9 Cigarettes	21.3
		10–19 Cigarettes	41.5
		20+ Cigarettes	12.7
N	9324

The time period between the base year and the last year was rather long: 14 years. This long survey period is important for research on smoking because smoking is addictive, meaning that the within-individual variation of smoking is small during a short period. When this variation is small, it is difficult to precisely estimate *β*_1_ because identification in fixed effects models entirely relies on the variation. Inertia in smoking status is more severe in developing countries because people there are less conscious of and concerned with the health hazards of smoking.

Table 2 illustrates the importance of a long panel. The correlation coefficient of smoking status in 1993 and 1997 was as high as 0.71, despite 4 years apart. When the time gap was 3 years between 1997 and 2000, the coefficient was slightly greater at 0.76. Even 7 years apart, the correlation coefficient of smoking status between 1993 and 2000 was almost the same as that between 1993 and 1997. Between 1993 and 2007, the correlation coefficient of smoking decreased to 0.58. This coefficient was not low in value, but low enough to precisely estimate *β*_1_.

**Table 2:** Correlation coefficients of smoking status over time

	Smoke in 1993	Smoke in 1997	Smoke in 2000	Smoke in 2007
Smoke in 1993	1.00			
Smoke in 1997	0.71	1.00		
Smoke in 2000	0.68	0.76	1.00	
Smoke in 2007	0.58	0.66	0.69	1.00

Recall that *β*_1_ was estimated from a change in smoking status or smoking intensity. If the change was mainly from smoking to nonsmoking, *β*_1_ would show only the effects of quitting, not of smoking, on weight-related outcomes. The transition rates in smoking status between 1993 and 2007 in Table 3, however, demonstrate that there were enough transitions between smoking and nonsmoking to argue that *β*_1_ also estimated the effects of smoking on weight-related outcomes. For example, 23.0 % of nonsmokers in 1993 were smokers in 2007, and 15.9 % of smokers in 1993 were nonsmokers in 2007.

**Table 3:** Transition between smoking and nonsmoking in 1993 and 2007

	Nonsmoking in 2007	Smoking in 2007	Number
Nonsmoking in 1993	490 (77.0 %)	146 (23.0 %)	636
Smoking in 1993	269 (15.9 %)	1426 (84.1 %)	1695
N	759	1572	2331

### Results for smoking status

Table 4 presents the effects of smoking on weight. When we entered basic demographics along with smoking status, *β*_1_ indicated a negative effect of smoking on weight (Column 1). If a man became a smoker, his weight decreased by about 0.9 kg. Alternatively, if he quit smoking, he gained weight by that amount. When we added two variables related to earnings (Column 2), *β*_1_ only slightly changed. This small change is consistent with the contrasting effects of earnings on weight via smoking. That is, in Indonesia, as in other developing countries, weight and earnings are positively correlated [20, 27, 33]—Column 2 also confirms this. Given this, higher earnings may increase one’s awareness of the health hazards of smoking and help him quit smoking. Conversely, higher earnings enable a man to purchase more cigarettes. We obtained the same patterns when we used OLS (Columns 3 and 4). More importantly, *β*_1_ estimated by OLS was more than three times as great as that estimated by fixed effects models.

**Table 4:** Effects of smoking status on weight

	Fixed effects		OLS	
	1	2	3	4
Smoke	−0.918 (0.213)***	−0.978 (0.219)***	−3.084 (0.327)***	−2.989 (0.319)***
Age	0.651 (0.062)***	0.618 (0.062)***	0.492 (0.070)***	0.395 (0.068)***
Age^2^ (/100)	−0.657 (0.055)***	−0.623 (0.055)***	−0.598 (0.076)***	−0.469 (0.074)***
Height	0.351 (0.051)***	0.358 (0.052)***	0.700 (0.026)***	0.674 (0.025)***
Married	0.568 (0.691)	0.560 (0.691)	2.317 (0.939)**	2.303 (0.947)**
Separated/Divorced/Widowed	0.418 (0.761)	0.386 (0.767)	0.678 (1.107)	1.075 (1.114)
Urbanity	0.215 (0.332)	0.191 (0.338)	2.894 (0.322)***	2.095 (0.326)***
Ln(Monthly Earnings)		0.289 (0.060)***		1.317 (0.111)***
Zero Earnings		1.211 (0.307)***		5.582 (0.566)***
Constant	−16.67 (8.16)**	−18.20 (8.41)**	68.50 (4.45)***	−68.09 (4.40)***
Year fixed effects	Yes	Yes	Yes	Yes
N	9320	9022	9320	9022
Overall R^2^	0.260	0.286	0.325	0.347

We applied longitudinal weights. Robust standard errors are in parentheses. For OLS results, we clustered standard errors at the individual level. *:*p*-value <0.10; **:*p*-value <0.05; ***:*p*-value <0.01

Panel A in Table 5 presents the results when we considered BMI for *w*. We included covariates identical to those in the corresponding column of Table 4, except that we excluded height because BMI contains height. In any event, *β*_1_ hardly changed whether we included or excluded height (not shown). For brevity, we listed only *β*_1_, but the overall results were the same as before. Because weight constitutes a numerator in BMI, it is expected that the sign of the coefficient on BMI would be the same as that on weight in Table 4. All columns in Table 5 confirm this expectation. A more interesting point is the size. The size of *β*_1_ was small, as for weight: smokers’ BMI was only 0.34–0.37 (about 1.6 % of the mean BMI) less than that of nonsmokers (Columns 1 and 2). Other main results were qualitatively the same as for Table 4; controlling for earnings-related variables only slightly changed *β*_1_ (Column 2), and OLS greatly overestimated the effects of smoking on BMI vis-à-vis fixed effects models—more than three times (Columns 3 and 4).

**Table 5:** Effects of smoking status on BMI and obesity

	Fixed effects		OLS	
	1	2	3	4
Panel A: BMI				
Smoke	−0.348 (0.082)***	−0.369 (0.084)***	−1.187 (0.126)***	−1.150 (0.123)***
Overall R^2^	0.045	0.089	0.106	0.136
Panel B: BMI ≥25				
Smoke	−0.018 (0.011)	−0.022 (0.012)*	−0.101 (0.014)***	−0.098 (0.014)***
Overall R^2^	0.005	0.015	0.068	0.092
Demographics	Yes	Yes	Yes	Yes
Earnings Covariates	No	Yes	No	Yes
Year Fixed Effects	Yes	Yes	Yes	Yes
N	9320	9022	9320	9022

We controlled for covariates identical to those in the corresponding columns of Table 4, except that we excluded height. We applied longitudinal weights. Robust standard errors are in parentheses. For OLS results, we clustered standard errors at the individual level. *:*p*-value <0.10; ***:*p*-value <0.01

Panel B presents the results when we considered obesity for *w*; the covariates were the same as for BMI. When we excluded the earnings-related variables, *β*_1_ was not statistically significant (Column 1 of Panel B). When we included the earnings-related variables, *β*_1_ became only weakly significant (Column 2 of Panel B). Despite the weak statistical significance, it could be of interest, if the size was large. *β*_1_ indicates that smokers were 2.2 percentage points less likely to be obese than nonsmokers, which is 16.2 % of the mean. The size is non-negligible, but the evidence was not compelling because the estimation was not precisely done. Comparisons of results derived from the two models show the same patterns as before: OLS produced much greater *β*_1_. This time, the difference was 4.5–6 times as great as that estimated by fixed effects models. Because *β*_1_ estimated by fixed effects models was not statistically significant at conventional levels, we cannot place much emphasis on the exact sizes of the differences.

### Results for smoking intensity

When we considered smoking intensity in linear form, *β*_1_ estimated by fixed effects models was statistically nonsignificant with a very high p-value, regardless of the dependent variables; this remained true whether or not we treated nonsmokers with a dummy variable. Including the earnings-related variables did not affect the results in any way (not shown).

It is strange that smoking itself was statistically significantly related to weight and BMI, but smoking intensity was not. Figure 1 suggests that the main action between smoking and weight-related outcomes may take place with a smoking intensity of less than a pack per day. Furthermore, evidence suggests that smoking produces a number of biological mediators of inflammation through its effect on immune-inflammatory cells, which results in an immunosuppressant state [34]. In turn, chronic inflammation is a risk factor for metabolic disorders, including obesity [35]. Therefore, the putative negative relationship between smoking and weight is opposed by the positive relationship in varying strength at varying points. These reasons suggest that the relationship between smoking intensity and weight-related outcomes may be nonlinear. We thus entered the series of dummy variables for smoking intensity—0, 1–9, 10–19, and 20+ cigarettes consumed per day—into specification (1), along with the full set of covariates (those in Column 2 of Table 4). For brevity, Table 6 lists only the coefficients on the dummy variables. The results confirm that the relationship was nonlinear: the greatest relationship was estimated for 10–19 cigarettes, instead of 20+ cigarettes. Nevertheless, the overall patterns were the same as those for smoking status.

**Table 6:** Effects of smoking intensity on weight-related outcomes

	1	2	3
Panel A: fixed effects	Weight	BMI	BMI ≥25
0 cigarette	Reference	Reference	Reference
1–9 cigarettes	−0.437 (0.207)**	−0.177 (0.079)**	−0.027 (0.012)**
10–19 cigarettes	−0.695 (0.200)***	−0.275 (0.077)***	−0.024 (0.012)*
20+ cigarettes	−0.273 (0.257)	−0.114 (0.099)	−0.012 (0.015)
Overall R^2^	0.275	0.057	0.048
Panel B: OLS			
0 cigarette	Reference	Reference	Reference
1–9 cigarettes	−2.162 (0.367)***	−0.838 (0.142)***	−0.077 (0.016)***
10–19 cigarettes	−2.906 (0.343)***	−1.129 (0.133)***	−0.099 (0.015)***
20+ cigarettes	−2.126 (0.449)***	−0.824 (0.172)***	−0.070 (0.019)***
R^2^	0.341	0.129	0.088

The sample size was 9022. We controlled for covariates identical to those in Column 2 of Table 4 but excluded height for Columns 2 and 3. We applied longitudinal weights. Robust standard errors are in parentheses. For OLS results, we clustered standard errors at the individual level. *:*p*-value <0.10; **:*p*-value <0.05; ***:*p*-value <0.01

## Discussion

We acknowledge that the IFLS does not contain information on fat and fat distribution. This information is important because even if smokers lose weight, they may gain abdominal fat, which is a more accurate measure of harm to health. That said, we argue that a lack of this information does not dramatically change our main argument because the effects of smoking on weight-related outcomes were consistently small for all the outcomes. Even if changes in smoking behavior change the distribution of fat, the small effects of smoking suggest that the change in abdominal fat is unlikely to be dramatic enough to pose a serious health threat.

Researchers have typically correlated smoking and weight-related outcomes, thereby failing to estimate the causal effects of smoking on weight-related outcomes. Recently, some health economists tried to address this issue by using IVs, but their results were not robust to specification changes. As a powerful alternative method, we employed fixed effects models. The most important feature of this method for our purposes is to control for time invariant individual heterogeneity. Methods based on correlation, notably OLS, cannot address this concern. We demonstrated the importance of controlling for it by comparing results derived from both methods. Although fixed effects models cannot address reverse causality and bias stemming from time-varying individual heterogeneity, we argue that they were probably negligible. Therefore, a fixed effects model provides a valuable alternative that estimates the effects of smoking on weight-related outcomes—possibly close to causality.

Our main finding is that estimation precision notwithstanding, the influence was very small: a quitter would gain weight by at most 1 kg. The results were similar for BMI and obesity. These small sizes of potentially overestimated effects strengthen our argument that the causal effects of smoking on weight are small. At the same time, our results demonstrate that ignoring time invariant individual heterogeneity results in great overestimates of the effects. Therefore, some previous findings of the large effects of smoking on weight-related outcomes might result from the failure of controlling for individual heterogeneity.

One may wonder why the effects of smoking on weight-related outcomes are so small. Given that we detected a nonlinear relationship between smoking intensity and weight-related outcomes in Table 6, it could be that smoking generates two contrasting effects. One is that smoking increases physical metabolism and reduces consumption of sweet food; the other is that chronic inflammation caused by smoking increases weight. The negative relationship is well recognized, but the positive one is not. Moreover, the fact that no study has found a positive relationship between smoking and weight suggests that the negative relationship is stronger than the positive one, but, according to our results, only slightly so. Based on this speculation, future research can determine the exact mechanisms, while focusing on the positive relationship as well.

Our results are based on data derived from a developing country but in line with the current knowledge in the US. Chou et al. initiated this line of research in health economics, using repeated cross-sections from Behavioral Risk Factor Surveillance System data, augmented by other data [4]. However, they focused on the causal effects of cigarette prices (not smoking per se) on weight in a reduced form model. Analyzing the same data, Gruber and Frakes used IVs and found that people who quit smoking were 56 % *less* likely to be obese; as they admitted, however, the size was implausibly large [6]. At the same time, they argued that the sizes of Chou et al.’s estimates were similar to theirs with the opposite sign, casting doubt on the results of both studies. Chou et al. responded that the sizes of their estimates were reasonable, but this rejoinder still focused on the effects of cigarette prices on weight loss [5].

Nonnemaker et al. reconciled these contrasting results [7]. They pointed out that the key lay in controlling for state-specific time trends by demonstrating that the trends were correlated both with state cigarette costs (prices or taxes) and obesity and this led to a spurious relationship between cigarette costs and obesity; the real effects identified in this study were very small. Using the same idea but different data (the National Longitudinal Study of Youth 1979) and methods, Baum agreed with Nonnemaker et al. [8]. Specifically, he controlled for the trends by creating comparison and treatment groups; the control group was not affected by state cigarette costs, whereas the treatment group was affected by the costs. The results suggested that types of cigarette taxes were immaterial once state-specific time trends were controlled for; the magnitudes of cigarette costs were also small.

Fang et al.’s study is of interest because they examined a developing country, namely, China [9]. Their estimation strategy for causality was based on IVs, which also partially relied on cigarette costs. Their results suggested that if an average male smoker quit smoking (a reduction of 16.8 cigarettes per day), his BMI would increase by about two. The size of the effect was considerably larger than that suggested by medical research. Note that it took almost two decades even during the “obesity boom” in the US in 1976–1994 for BMI to increase by 1.5 [36]. The large magnitude argued by Fang et al. could be attributed to the IVs: the coefficient on the number of cigarettes per day with the IVs was more than six times as great as that without the IVs. Although they argued that the IVs corrected for endogeneity, the difference seems too large, casting doubt on the exclusion restrictions of their IVs. This implausibility supports our empirical strategies.

## Conclusions

Our Indonesian sample consisted of over 9000 men aged 15–55 years; each of them was observed in 1993, 1997, 2000 and 2007. We applied fixed effects models and precisely estimated the effects of smoking on weight, BMI, and obesity in Indonesia. We found that smoking reduced weight-related outcomes and the effect was statistically significant. However, the size was practically nil. Our evidence suggests that the great effects reported in the literature might be driven by bias stemming from time invariant individual heterogeneity. Because Indonesia exhibits smoking-related conditions typical in developing countries, our results for Indonesia may be generalized to other developing countries.

## Abbreviations

BMI: body mass index
EA: enumeration area
OLS: ordinary least squares
IFLS: Indonesian family life survey
IV: instrumental variable
WHO: the World Health Organization
